# Modeling Electrochemical Impedance Spectroscopy Using Time-Dependent Finite Element Method

**DOI:** 10.3390/s24227264

**Published:** 2024-11-13

**Authors:** Yawar Abbas, Laura van Smeden, Alwin R. M. Verschueren, Marcel A. G. Zevenbergen, Jos F. M. Oudenhoven

**Affiliations:** imec at Holst Centre, 5656 AE Eindhoven, The Netherlands; laura.vansmeden@imec.nl (L.v.S.); alwin.verschueren@imec.nl (A.R.M.V.); marcel.zevenbergen@imec.nl (M.A.G.Z.); jos.oudenhoven@imec.nl (J.F.M.O.)

**Keywords:** finite element method, electrochemical impedance spectroscopy, COMSOL model, time-dependent analysis, simulations

## Abstract

A time-dependent electrochemical impedance spectroscopy (EIS) model is presented using the finite element method (FEM) to simulate a 2D interdigitated electrode in an aqueous NaCl electrolyte. Developed in COMSOL Multiphysics, the model incorporates ion transport, electric field distribution, Stern layer effects, and electrode sheet resistance, governed by the Poisson and Nernst–Planck equations. This model can predict the transient current response to an applied excitation voltage, which gives information about the dynamics of the electrochemical system. The simulation results are compared with the experimental data, reproducing key features of the measurements. The transient current response indicates the need for multiple excitation cycles to stabilize the impedance measurement. At low frequencies (<1 kHz), the voltage drop at the Stern layer is significant, while at higher frequencies (>100 kHz), the voltage drop due to sheet resistance dominates. Moreover, the amplitude of the excitation voltage influences the EIS measurement, higher amplitudes (above 0.1 V) lead to non-linear impedance behavior, particularly at low ion concentrations. Discrepancies at low frequencies suggest that Faradaic processes may need to be incorporated for improved accuracy. Overall, this model provides quantitative insights for optimizing EIS sensor design and highlights critical factors for high-frequency and low-concentration conditions, laying the foundation for future biosensing applications with functionalized electrodes.

## 1. Introduction

Electrical impedance spectroscopy (EIS) has emerged as a pivotal technique in electrochemical sensing due to its non-invasive and label-free capabilities [1,2,3,4]. This method facilitates the analysis of an electrochemical system subjected to an applied voltage or current between working and counter electrodes. EIS is instrumental in assessing the properties of both electrode surfaces and bulk electrolytes across various electrochemical sensing applications [5,6].

The technique’s utility spans a wide frequency range, providing distinct insights into the electrochemical system. At low frequencies (below ~100 Hz), EIS is particularly adept at probing the electrode–electrolyte interface, giving vital information about electric double-layer capacitance (EDLC) [5,6,7]. Conversely, at higher frequencies (above ~1 kHz), the impedance response is predominantly influenced by the electrolyte resistance, as the effects of the double-layer capacitance diminish. This frequency-dependent behavior underscores the importance of understanding both the electrode–electrolyte interface and the bulk electrolyte properties in optimizing sensor performance.

Finite element modeling (FEM) has become an essential tool for simulating electrochemical systems, enabling the exploration of various design configurations and system behaviors under different conditions, including geometry, surface morphology, material properties, and ambient conditions [8,9]. COMSOL Multiphysics^®^ is frequently used for FEM simulations, allowing the integration of multiple physical phenomena, such as electrostatics and electrochemical processes. While simplified models like the Randles equivalent circuit provide a basic framework for understanding impedance behavior, they often fall short in complex geometries and non-linear, time-dependent scenarios [10].

**Figure 1:**
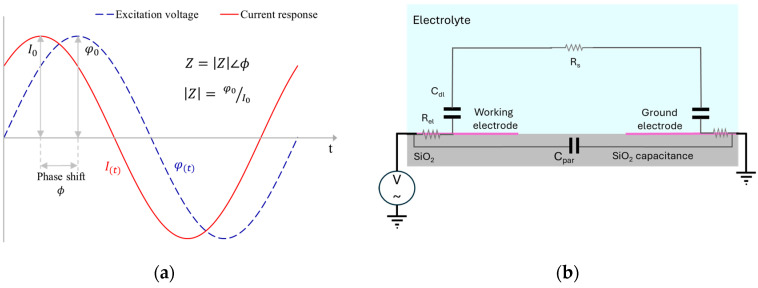
(**a**) The voltage excitation signal and current response to evaluate the impedance at a particular frequency. (**b**) Schematic of the equivalent electrical components and elements for the impedance electrode in an electrolyte for a planar two-electrode system.

EIS functions by perturbing an electrochemical system using a sinusoidal signal, such as an alternating current (AC) excitation potential, $\varphi_{\left(t\right)}=\varphi_{0}Sin(\omega t)$, over a broad frequency range. The system’s response (if within the linear regime) is measured as a corresponding sinusoidal current generated, $I_{\left(t\right)}=I_{0}Sin(\omega t+\phi )$, with a possible phase shift ϕ. According to Ohm’s law, the magnitude of the impedance is determined by the ratio of the voltage amplitude to the current response amplitude, while the phase shift corresponds to the phase difference between the peaks of the voltage and current responses, as shown in Figure 1a.

Recent literature has reported numerous modeling efforts for EIS-based sensors across diverse applications, including fuel cells, biosensing, and environmental monitoring [11,12,13,14]. Most studies have focused on stationary conditions, but only few have explored time-dependent analyses (the transient period before the system turns stationary). One exception is a study on fuel cells using mixed ionic–electronic conducting (MIEC) materials [12]. These investigations highlight the significance of transient response analysis in understanding the kinetics of electrochemical reactions and the effects of rate-limiting steps. However, time-dependent studies focusing on polarizable electrodes in aqueous electrolytes—particularly concerning ion transport dynamics and transient phenomena at the electrode–electrolyte interface, including the behavior of the Stern and Debye layers—remains largely unexplored.

In this study, the development of a time-dependent model utilizing COMSOL Multiphysics 6.2 (COMSOL AB, Stockholm, Sweden) to simulate the EIS response of a planar interdigitated electrode sensor is presented. This model aims to compute ion transport resulting from an applied excitation voltage, for a monovalent salt solution (e.g., NaCl) at varying concentrations. The key aspects under investigation include the transient current response, Debye layer analysis, the influence of applied excitation voltage, and impedance evaluation at various excitation frequencies. Experimental validation is performed using interdigitated electrodes made of bare platinum, with EIS plots derived from experimental data compared against the simulations. Future work will involve functionalizing these electrodes with bioreceptors to enhance selectivity, expanding the applicability of the model in sensing technologies.

The details of all the symbols used in this paper for equations are given in Table 1. This article is a revised and expanded version of another paper, entitled ‘Time-dependent modeling of an electrochemical impedance spectroscopy-based sensor’, which was presented at the ‘11th International Electronic Conference on Sensors and Applications’ on 26 to 28 of November 2024 [15].

## 2. Materials and Methods

In this study, an interdigitated platinum electrode pair on a silicon substrate, featuring a SiO_2_ isolation layer, is utilized to validate the COMSOL model, as illustrated schematically in Figure 1b. The model incorporates the parasitic effects introduced by the 500 nm thick SiO_2_ layer, which contributes to an additional parasitic capacitance, C_par_. Furthermore, the sheet resistance of the planar electrode, R_el_, is also accounted for in the model to ensure an accurate representation of the system’s electrical behavior.

When analyzing such electrochemical system in a time-dependent analysis, the dynamic behavior can be analyzed. One of these behaviors is the spatial profile of the ions near the electrode surface over time, consequently giving information on the buildup of the Debye layer. The Debye layer thickness, $x_{D}$, can also be determined theoretically, $x_{D}=\sqrt{\epsilon_{liq}RT/2F^{2}c_{bulk}}$, for a monovalent symmetric electrolyte [16]. The theoretical calculation of the Debye layer thickness assumes stationary conditions, neglecting time-dependent changes, polarization effects, and non-ideal behavior. In contrast, a finite element method (FEM) model can simulate dynamic ion behavior, account for complex geometries, electrochemical reactions, and non-linear effects, providing a more accurate and detailed analysis of the Debye layer formation over time.

### 2.1. Model Equations

The COMSOL model is developed as a two-dimensional framework to simulate the voltage drop and ion concentrations within an electrolyte. To solve for both the voltage distribution and ion concentrations across the domain, two governing equations are employed: the Poisson equation, given in Equation (1), and the Nernst–Planck equation, given in Equation (2) [17]. Moreover, the conservation of charged species and the dynamics of ion concentration, given in Equation (3), is implemented.

(1) $$\nabla^{2}\varphi +\frac{F\sum_{i}z_{i}c_{i}}{\epsilon_{liq}}=0$$

(2) $$J_{i}=-D_{i}\nabla c_{i}-\frac{D_{i}z_{i}Fc_{i}\nabla \varphi }{RT}$$

(3) $${\frac{\partial c_{i}}{\partial t}=-\nabla \cdot J}_{i}+R_{i}=D_{i}\nabla^{2}c_{i}+\frac{D_{i}z_{i}F{\nabla \cdot (c}_{i}\nabla \varphi )}{RT}+R_{i}$$

The Stern layer, which constitutes the innermost region of the electrical double layer (EDL), is typically composed of ions with charges opposite to that of the surface, electrostatically bound to it. These adsorbed ions exhibit no relative motion with respect to the surface due to the strong adsorption forces. In contrast, ions within the diffuse layer possess greater mobility, as they are not as strongly bound to the surface. Additionally, the sheet resistance of the electrode introduces a resistive element at the electrode surface, which is incorporated into the model as well. These factors collectively define the boundary conditions at the electrode. At the working electrode boundary, the potential is expressed as the excitation voltage minus the voltage drop resulting from the sheet resistance ($\frac{d_{el}}{\sigma_{el}}$) and the Stern layer thickness ($\lambda_{S}$). This is similar at the ground electrode, except for the absence of the excitation voltage. In these boundary equations, the term $\frac{{\epsilon_{liq}d}_{el}}{\sigma_{el}}$ is the coefficient of the sheet resistance drop and denoted as $k_{el}$, whereas the term $\frac{\epsilon_{liq}{\cdot \lambda }_{S}}{\epsilon_{S}}$ is the coefficient of the Stern layer drop and denoted as $k_{s}$. Both coefficients have different SI units (see Table 2) and are fitted to experimental data to determine their values, which are subsequently verified for physical consistency.

The equations for the boundary condition at the working and the ground electrodes are given in Equations (4) and (5).

(4) $${\varphi_{y=0}=\varphi }_{\left(t\right)}-\frac{\epsilon_{liq}d_{el}}{\sigma_{el}}\cdot \frac{d\nabla \varphi \cdot \vec{n}}{dt}-\frac{\epsilon_{liq}\lambda_{s}}{\epsilon_{s}}\cdot \nabla \varphi \cdot \vec{n}$$

(5) $$\varphi_{y=L}-\frac{\epsilon_{liq}d_{el}}{\sigma_{el}}\cdot \frac{d\nabla \varphi \cdot \vec{n}}{dt}-\frac{\epsilon_{liq}\lambda_{s}}{\epsilon_{s}}\cdot \nabla \varphi \cdot \vec{n}$$

### 2.2. COMSOL Model Details

The system’s parameters and initial conditions, outlined in Table 2, must be specified at the outset. These parameters are used in both the domain equations and boundary conditions. To investigate the effect of bulk ion concentration, three distinct salt concentrations—1 mM, 10 mM, and 100 mM—are utilized. A sinusoidal voltage excitation with an amplitude of 10 mV is applied, accompanied by a frequency sweep ranging from 0.1 Hz to 1 MHz.

**Figure 2:**
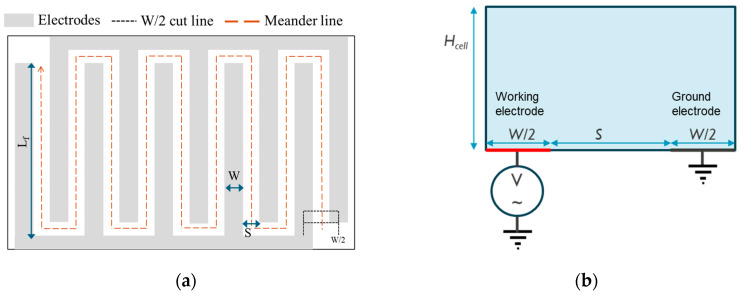
(**a**) Schematic representation of the IDE design for sensor validation in the laboratory. (**b**) Approximation of the IDE configuration adapted for a 2D geometry in COMSOL simulations.

The design of the interdigitated electrodes (IDEs) used for model validation is depicted in Figure 2a. Modeling such a geometry and scale in 3D would require substantially greater computational time and resources. To optimize this, a 2D approximation of the IDE design is utilized. This is achieved by considering the cross-section of half the electrode width from the first electrode finger at one end of the IDEs and then aligning the meander spacing between them. The IDEs are approximated as two parallel planar electrodes, with an electrode spacing, S, electrode width W/2 (half the width of one electrode finger), and a length equal to the total meander spacing, L_m_. This 2D representation of the parallel planar electrodes is shown in Figure 2b, where the length L_m_ is later accounted to scale the current response. This approximation of the meandering towards the parallel electrode may introduce inaccuracies, as it neglects the fringing effects of the electric field. The values of all relevant model dimensions are provided in Table 3.

The model focuses on solving two key variables: the electrolyte potential (*φ*) and ion concentration (*c_i_*). The Poisson equation (Equation (1)) describes the relationship between the electrolyte potential and the spatial distribution of charge, which arises from ion concentrations. Ion transport in the electrolyte, driven by the applied excitation voltage, is governed by the Nernst–Planck Equation (2), which accounts for both diffusion and migration processes. To determine the time-dependent evolution of ion concentrations, the law of species conservation (Equation (3)) is used, linking the ion flux to changes in concentration over time.

These governing equations are implemented within a time-dependent simulation framework in COMSOL, utilizing two physics modules: electrostatics (es) and transport of dilute species (tds). The electrostatics module solves for the electrolyte potential, while the transport of dilute species module addresses the dynamics of ion concentrations. In the es module, boundary conditions are defined for both the working electrode and the ground electrode. The tds module is responsible for the ion properties, such as charge number, diffusion coefficient, and bulk concentration in the electrolyte. An excitation voltage, *φ(t)*, with a specified amplitude and frequency, is applied to the working electrode. It is assumed that the electrode surface is at its point of zero charge before the excitation voltage is applied. However, it is recognized that an intrinsic surface charge typically exists when the electrode is immersed in the electrolyte, even in the absence of an external voltage, which may lead to discrepancies compared to experimental results.

At the electrode–electrolyte interface, the ion and voltage distributions evolve as the Debye layer forms, a process occurring within a sub-micrometer distance from the electrode surface. To accurately resolve this phenomenon, the mesh size in the simulation must be fine enough to capture the behavior of the Debye layer. For low ion concentrations, such as 1 mM, the Debye length is approximately 1 nm, necessitating a mesh size smaller than this value. The mesh size close to the electrode surface is reduced in an iterative way unless no change in the resulting current response is observed. In this simulation, a mesh size of 0.01 nm (normal to the electrode surface) near the electrode surface is taken. A mapped meshing technique is used with the non-linear distribution of the mesh size to have very fine mesh close to the electrode surface (0.01 nm) and coarse mesh far from electrode surface; e.g., at a 1 µm distance from the electrode, the mesh size would be 0.12 µm.

The time-dependent study applies three periods of sinusoidal excitation voltage to track the evolution of the potential and ion concentration in the electrolyte over time. In cases where multiple excitation frequencies are considered, a parametric sweep from 0.1 Hz to 1 MHz is conducted to analyze the frequency-dependent behavior of the system.

Upon completion of the simulation, the potential (*φ*) and the electric field (−*∇φ*) within the electrolyte are evaluated to compute the current response and, ultimately, the system’s impedance. The current response is determined by calculating the surface integral of the electric field normal to the electrode surface, as described in Equation (6), where A represents the surface area of the working electrode. The impedance, Z, is characterized in terms of its magnitude, ∣Z∣, and phase shift, both of which are depicted using Bode plot representation. The magnitude of the impedance is determined, as mentioned in Figure 1a, during the third period of the applied excitation. In this same cycle, the phase shift is calculated by measuring the time difference, Δt, between the peak of the excitation voltage and the corresponding peak of the current response. This phase shift provides insight into the system’s dynamic behavior and is a key parameter in analyzing the electrochemical response.

(6) $$I_{\left(t\right)}=∯\epsilon_{liq}\frac{d\nabla \varphi \cdot \vec{n}}{dt}\cdot dA$$

### 2.3. Experimental

**Figure 3:**
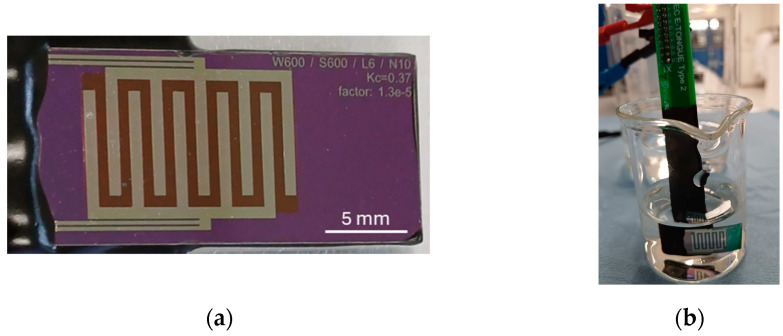
(**a**) IDE sensor fabricated on a silicon substrate utilized for laboratory validation. (**b**) IDE sensor immersed in an aqueous electrolyte during laboratory measurements.

To validate the model, EIS measurements were performed. The experimental setup used IDEs with 5 fingers per electrode, as shown in Figure 3a, made of platinum on a silicon substrate coated with SiO_2_. The measurements were carried out using a MultiPalmSens4 device and controlled by the software MultiTrace 4.5 from PalmSens BV (Houten, the Netherlands). The system was configured to operate in potentiostatic mode with a current range between 1 nA and 10 mA, with the direct current potential (E_dc_) set at 0.0 V and an alternating current potential (E_ac_) of 10 mV amplitude, without pre-treatments.

The EIS data were collected by performing a frequency sweep ranging from 1 MHz to 0.1 Hz. A total of 71 frequencies were measured, with 10 points per decade (total duration of 3.2 min), to provide detailed frequency-dependent information about the system’s impedance.

The electrodes were fully immersed in NaCl solutions of varying concentrations as illustrated in Figure 3b. NaCl (Acros Organics, Geel, Belgium; >99.5% purity; CAS 7647-14-5) was dissolved in deionized water to make a stock solution of 1 M NaCl, which was used to prepare a 10-time dilution series of 100, 10, and 1 mM NaCl, respectively. Three different NaCl concentrations were tested: 1 mM, 10 mM, and 100 mM, all at room temperature (20–21 °C). To ensure measurement accuracy, the electrodes were rinsed thoroughly with deionized water between each test to eliminate any residual contamination from the previous solution.

## 3. Results

### 3.1. Transient Response

**Figure 4:**
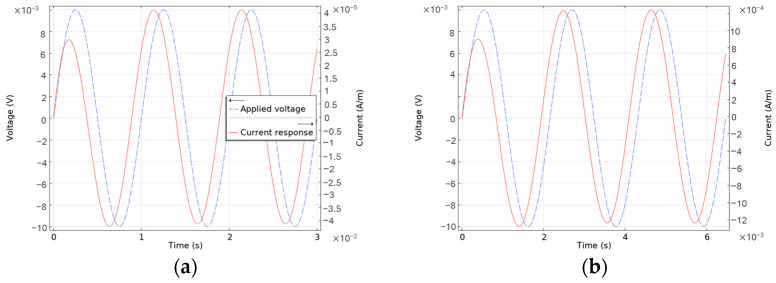
(**a**) Plots showing the applied voltage and the current response of (**a**) 1 mM NaCl electrolyte at 100 Hz and (**b**) 1 mM NaCl electrolyte at 464 Hz.

A time-dependent analysis was performed over three periods of voltage excitation to capture the system’s behavior across a range of frequencies, from 0.1 Hz to 1 MHz. The voltage and current responses over time for 1 mM and 100 mM ion concentrations at 100 Hz and 464 Hz are illustrated in Figure 4a and Figure 4b, respectively. The selected frequencies are near the cut-off frequency for both concentrations, i.e., 100 Hz for 1 mM and 464 Hz for 100 mM ion concentrations. The cut-off frequency is the frequency at which the phase shift between the applied excitation voltage and the resulting current reaches 45 degrees. This marks the transition between the capacitive (double-layer capacitance) and the resistive (electrolyte resistance) behavior in the electrochemical system.

### 3.2. Debye Layer Formation

**Figure 5:**
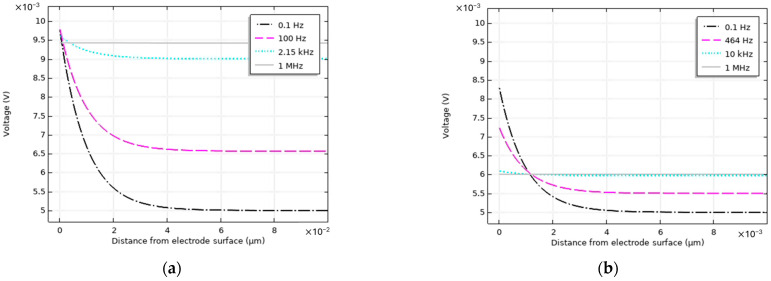
(**a**) Spatial profile of the voltage near the working electrode at various excitation frequencies (**a**) for 1 mM salt concentration and (**b**) for 100 mM salt concentration.

In the transient analysis, as mentioned before, it is possible to observe the dynamics of the electrochemical system, specially at the electrode–electrolyte interface. For example, it is possible to observe the buildup of the Debye layer or ion profile near the electrode surface at various frequencies and at specific moments in time. The computed voltage profiles near the electrode surface for various frequencies at the moment of the voltage peak (one-fourth of the period) where the applied potential value is 10 mV for 1 mM and 100 mM ion concentrations is shown in Figure 5a and Figure 5b, respectively. These frequencies are chosen to show interesting regions, i.e., 0.1 Hz (lowest frequency), 100 Hz (near the cut-off frequency for 1 mM concentration), 464 Hz (near the cut-off frequency for 100 mM concentration), 2.15 kHz (frequency of the lowest phase shift for 1 mM), 10 kHz (frequency of the lowest phase shift for 100 mM), and 1 MHz (highest frequency). Moreover, the x-axis is adjusted to show the voltage profile up to 10 times the theoretical Debye length. The theoretical Debye length, described in Section 2, for 1 and 100 mM ion concentrations are 9.63 nm and 0.96 nm, respectively.

### 3.3. EIS Results

**Figure 6:**
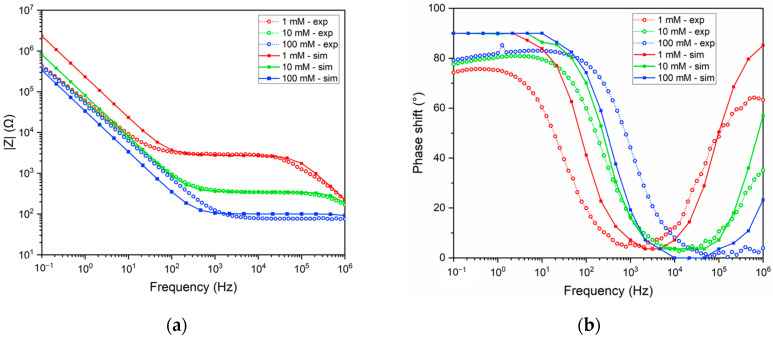
Bode plot of impedance as a function of excitation frequency for 1, 10, and 100 mM NaCl solutions obtained from laboratory experiments and simulations. (**a**) Impedance magnitude (|Z|) as a function of excitation frequency. (**b**) Phase angle as a function of excitation frequency.

The impedance response, including both the magnitude and phase shift, are derived from the simulated current and excitation voltage, following the post-processing described in COMSOL model details (Equation (6) and Figure 1a). The results are displayed in Figure 6, which shows the Bode plots for a frequency sweep ranging from 0.1 Hz to 1 MHz at different NaCl concentrations. The EIS data from lab experiments are also plotted; the simulation results are labeled as ‘sim’, while the experimental values are denoted as ‘exp’. Figure 6a and Figure 6b compare the magnitude of impedance and phase shift, respectively, for 1 mM, 10 mM, and 100 mM NaCl solutions.

### 3.4. Influence of the Amplitude of the Excitation Voltage

**Figure 7:**
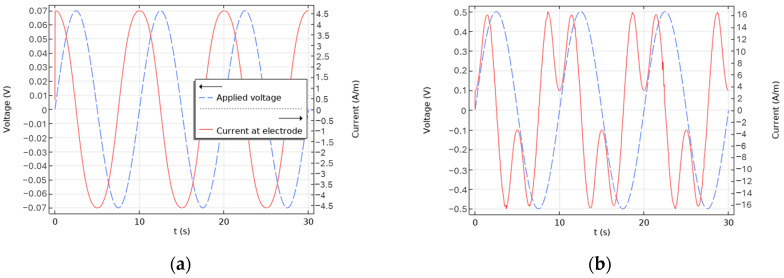
Applied voltage and current response for 1 mM concentration and 0.1 Hz at (**a**) 70 mV and (**b**) 0.5 V excitation voltage amplitude.

The amplitude of the excitation voltage signal is an important parameter and needs to be carefully chosen for reliable EIS measurements. Higher amplitudes can lead to non-linear behavior of the system. Various amplitudes of excitation voltage are applied (ranging from 10 mV to 0.5 V) to observe the effect on the current response and thus the resulting impedance. The transient current responses for 70 mV and 0.5 V amplitude applied potential are shown in Figure 7a,b. At higher amplitudes of the applied potential, the current signal becomes distorted and includes second and higher harmonic components. In lab experiments, the potentiostat only takes into account the first harmonic component of the current response with the same frequency as the excitation voltage, either by a lock-in technique or by a Fourier transformation. The magnitude of impedance is then determined by dividing the magnitude of applied voltage by the magnitude of current at the excitation frequency. To have experimental relevance, in our simulations, we use a similar technique to determine the magnitude of impedance to study the effect of excitation voltage amplitude.

**Figure 8:**
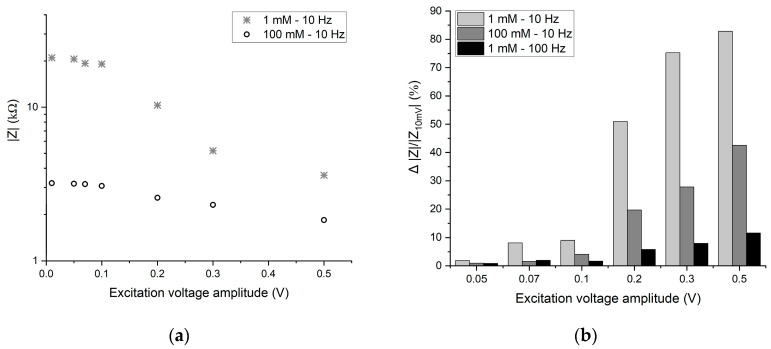
(**a**) Values of |Z| for 1 and 100 mM salt concentrations at various amplitudes of excitation voltage. (**b**) Relative deviation of the |Z| values for various amplitudes of excitation voltage as compared to |Z_10mV_| at 10 mV amplitudes of excitation voltage.

The |Z| values for 1 mM and 100 mM ion concentrations at various amplitudes of the excitation voltage are shown in Figure 8a. The deviation of the |Z| values as compared to the respective |Z| values evaluated at 10 mV excitation voltage amplitude (denoted as |Z_10mV_|) is depicted in Figure 8b. The effect of changing ion concentrations and the applied frequency on this deviation at various voltage amplitudes is evaluated.

## 4. Discussion

In Figure 4, the time-dependent behavior of the current in response to the applied excitation potential shows that the current peaks are lower in the initial period compared to the final period. This observation indicates that the system exhibits time-dependent characteristics and requires a finite duration to stabilize and reach a stationary condition. In both Figure 4a and Figure 4b, it took one period for the current response to reach a stationary state, i.e., 10 ms and 2.1 ms, respectively. During this process, the current signal undergoes a rapid transient phase before achieving a periodic steady state, at which point a constant phase shift with respect to the applied voltage is observed.

For accurate EIS measurements, it is crucial to determine the number of periods required for the current response to reach a periodic state before impedance estimation. This insight is essential for experimental setups, as a predefined number of excitation periods must be applied before impedance calculations can be performed reliably. Ensuring the system has reached steady-state conditions is vital to avoiding inaccuracies in impedance measurements.

Another advantage of time-dependent models is the insight they offer in terms of ion transport over time for various excitation frequencies resulting in insights in the dynamic of the electrode–electrolyte interface, such as the development of the Debye layer. The Debye layer characterizes how the electric potential decays from the electrode surface into the electrolyte. According to the Gouy–Chapman–Stern model, the potential as a function of distance x from the electrode surface decreases exponentially [19]. The Debye length is the distance over which the potential decays to about 1/e (1 exponent, i.e., approximately 37%) of its initial value at the surface [20]. In Figure 5a,b, the voltage profile of the working electrode for 1 mM and 100 mM is shown. At 0.1 Hz, it can be observed that the voltage profile indeed decreases exponentially and the distance from the electrode surface where the potential profile reaches 1/e of the potential at x = 0 is 9.94 nm for 1 mM and 0.93 nm for 100 mM ion concentrations. These values are in excellent agreement to the theoretical Debye layer values (0.96 nm and 9.63 nm for 1 and 100 mM ion concentrations, respectively). The decreasing-voltage profile of potential from the electrode surface decreases with the increase in frequency, e.g., at 1 MHz, the voltage profile is a straight horizontal line with a negligible gradient. Since with the increase in frequency, there is not enough time for ion transport to create an ion unbalance near the electrode surface, and thus, the Debye layer or ion segregation at the electrode surface diminishes with the increase in the excitation frequency.

**Figure 9:**
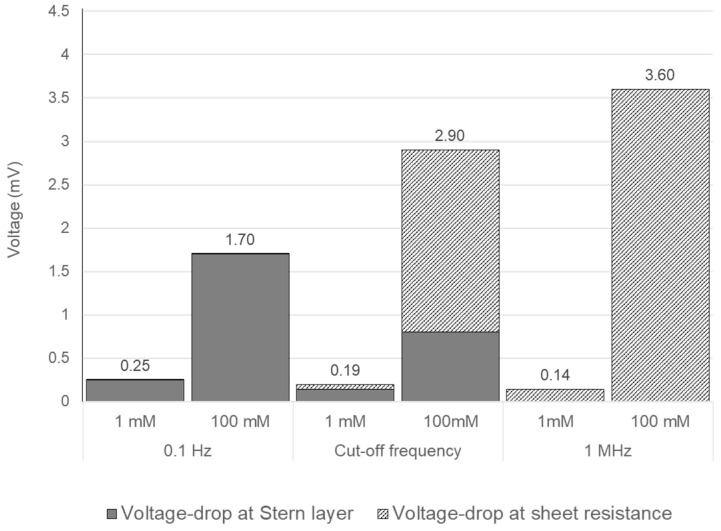
Voltage drop at the electrode surface due to the Stern layer and the sheet resistance for various excitation frequencies and ion concentrations.

In Figure 5a,b, the external voltage is 10 mV, but due to the Stern layer and sheet resistance drop at the electrode surface, lower potential values (than the 10 mV) are observed at the electrode surface (y = 0). This voltage drop is contributed by the Stern layer drop and the sheet resistance of the electrode. The total voltage drop at the working electrode and the contribution of the voltage drop due to Stern and sheet resistance for various excitation frequencies are given in Figure 9, all calculated at the moment of maximum excitation voltage. At low frequencies, such as 0.1 Hz, the drop at the Stern layer dominates and the voltage drop at the sheet resistance has negligible effect because the charging currents are low. With increasing ion concentration (from 1 to 100 mM), the Debye layers contain more charge, resulting in a stronger electric field and larger voltage drop over the Stern layer.

At high frequencies, such as 1 MHz, there is insufficient time for ion transport to build up a Debye layer, and consequently, the electric field and voltage drop over the Stern layers is negligible. However, at these frequencies, the current over the sheet resistance is highest (and conversely the absolute impedance is lowest, as in Figure 6a), resulting in a higher voltage drop over the sheet resistance. Moreover, at higher frequencies, the lower electrolyte resistance becomes comparable to the sheet resistance, especially for higher ion concentrations. This means, practically speaking, that it is important to consider the effect of sheet resistance at higher ion concentrations, as the total impedance—as a series connection of electrolytes and electrodes—cannot become smaller than the electrode resistance. Typically, sheet resistance values are in the order of 10 Ω per square [21]. At the cut-off frequency, for both 1 and 100 mM, there is an intermediate combined effect from both the voltage drop due to the sheet resistance and Stern layer.

The comparison of impedance magnitude (|Z|) and phase shift between simulated and experimental data, in Figure 6, for ion concentrations of 1, 10, and 100 mM reveals similar overall trends, though with some discrepancies. At 1 mM, both the simulated and experimental results indicate that the onset of the low-phase-shift plateau, occurring after the cut-off frequency, appears at approximately 1 kHz. At this plateau, the |Z| value is around 2.7 kΩ, signifying primarily resistive behavior, as confirmed by a near-zero phase shift. At frequencies exceeding 100 kHz, parasitic effects from the SiO_2_ layer become evident, leading to a phase shift increase, reaching approximately 85° at 1 MHz for the 1 mM concentration. Moreover, at higher frequencies, other parasitic effects such as electromagnetic noises can have an effect since the experiments were not performed in a Faraday cage, but in this model, this effect is not accounted for. For higher ion concentrations (10 and 100 mM), the influence of parasitic capacitance shifts to even higher frequencies, resulting in phase shifts of 56.9° and 23.3° at 1 MHz, respectively. This shift is attributed to the reduced electrolyte resistance at higher ion concentrations, which shortens the RC time constant in combination with the parasitic capacitance. Moreover, in Figure 6a, the impedance values at the low-phase-shift plateau, representing the electrolyte resistance, decrease with increasing ion concentration in both the simulated and experimental data. However, this decrease is not strictly proportional, especially at higher concentrations. For instance, the impedance decreases from approximately 2.7 kΩ at 1 mM to 0.34 kΩ at 10 mM, an eightfold reduction corresponding to a tenfold increase in concentration. However, between 10 mM and 100 mM, the impedance decreases only from 0.34 kΩ to 0.1 kΩ, a reduction by a factor of 3.4. This suggests that at higher concentrations, the |Z| plateau does not scale linearly with concentration and instead reaches a saturation point. This saturation is likely due to the increasing contribution of sheet resistance. Once the electrolyte resistance (R_s_) drops below 100 Ω, the sheet resistance (approximately 35 Ω) becomes significant. Therefore, when designing EIS sensors for high-concentration conditions, it is essential to consider the limiting effect of sheet resistance. This model is suited for polarizable electrodes typically from noble metals such as platinum, gold, and carbon. Moreover, as electrolyte NaCl is used, other electrolytes can also be simulated using their corresponding diffusion coefficient. Any reaction in the bulk electrolyte can be accounted by using the rate of reaction, R_i_.

At lower frequencies (<100 Hz), as shown in Figure 6a, the simulated impedance data exhibit consistent slopes of approximately -1 per decade across all concentrations. In contrast, the experimental data converge into a single curve at low frequencies (with a slope of approx. −1/decade) but show an offset when compared to the simulated results. This offset in |Z| is proportional to the electrolyte concentration; for example, at 0.1 Hz, |Z| increases by a factor of 2.4 when decreasing from 100 mM to 10 mM and by 2.8 when further reducing from 10 mM to 1 mM. This behavior suggests that, in the simulations, the electrical double-layer (EDL) capacitance is dominated by the concentration-dependent Debye layer, as expected. In this model, the Stern layer is treated as having a fixed thickness, independent of ion concentration, leaving the Debye layer to dominate at lower frequencies. Interestingly, in the experimental data, all concentrations converge to a similar |Z| value of approximately 420 kΩ, possibly due to Faradaic reactions or adsorption/desorption processes at the electrode surface, which are not captured by the COMSOL model. For future improvements in the model, a charge transfer phenomenon needs to be included either for a limited Faradaic reaction or the adsorption/desorption of ions.

At higher frequencies, specifically around 1 MHz, the effect of parasitic capacitance from the SiO_2_ layer on the silicon substrate becomes significant, as depicted in Figure 6b. At this frequency, the phase shift reaches approximately 23.3° for 100 mM and around 85° for 1 mM. This indicates that the parasitic capacitance from the SiO_2_ layer introduces a notable limitation, which must be considered when designing EIS-based sensors on silicon substrates.

In the phase shift plots for the simulated data (Figure 6b), the phase shift approaches 90° at low frequencies (<100 Hz) for all ion concentrations, indicating capacitive behavior. However, the experimental phase shift data deviate from this ideal behavior, with values lower than 90°—approximately 80° at 0.1 Hz—and a further decrease in phase shift as frequency decreases. This trend suggests the presence of additional mechanisms not accounted for in the current model at low frequencies, which will be addressed in future work.

So far, for all the simulations, an excitation voltage of 10 mV amplitude is used. For experimental EIS measurements, excitation voltage around this value is chosen, typically below 25 mV [22,23]. Whereas this value is typically selected by default, a higher amplitude would be beneficial to keep the signal-to-noise ratio higher; on the other hand, a higher voltage amplitude can result in a non-linear voltage–current relation. Therefore, a higher limit of the excitation voltage needs to be determined where the voltage–current is in linear regime. In Figure 7, it is evident that at higher amplitudes of excitation voltage, the current no longer remains in linear relation to the applied voltage and contains significant higher harmonics (notably at twice the excitation frequency). Moreover, the current value does not increase linearly to the increase in the voltage amplitude. As in Figure 7, for an approximately seven-time increase in the voltage amplitude (from 70 mV to 0.5 V), the current signal’s highest peak increases roughly 35-fold. The remarkable pattern (in Figure 7b) of double current peaks at high-voltage amplitudes can be explained as follows. First, notice that for the conditions in Figure 7a (f = 0.1 Hz and a low excitation amplitude of 70 mV), the electrode current is 90 degrees out of phase with the applied voltage, consistent with the Bode plot of Figure 6b. In that case, for every increase in the applied voltage, ions will be further separated, resulting in a positive polarization current. In Figure 7b, whenever the applied voltage exceeds the linear threshold (of about 0.1 V), the ions will drastically separate further, giving rise to an additional current peak. Similarly, for negative voltages decreasing below −0.1 V, an additional negative current peak is observed. Overall, this results in four current peaks per period of applied voltage: a second harmonic in current for non-linear voltage excitation. This means that with the increase in the amplitude of the excitation voltage, the measured |Z| value will decrease, even when other parameters for the system are kept constant. This is also evident in Figure 8a, where the |Z| decreases when the voltage amplitude increases above 0.1 V. The voltage–current response is in linear regime till 0.1 V; after that, |Z| decreases significantly for both 1 and 100 mM ion concentrations at 10 Hz. The decrease is sharp in the case of 1 mM; this means that for lower concentrations, the limitation of the applied amplitude is lower than at higher ion concentrations. This is also shown in Figure 8b, in that up to 0.1 V, the deviation from |Z_10mV_| (impedance at the lowest excitation amplitude, 10 mV) is within 10% for various concentrations and frequencies. Above 0.1 V, at 0.1 Hz, the deviation increases significantly, reaching 72 and 42% for 1 and 100 mM, respectively. Therefore, 0.1 V can be considered as an upper limit of the excitation voltage. The deviation at higher concentrations is lower, meaning for higher concentrations, such as 0.1 V, the excitation voltage can be increased further (between 0.1 and 0.2 V). Moreover, at higher frequencies, the effect of amplitude becomes smaller; at 0.5 V and 1 mM, the deviation for 10 and 100 Hz is 82 and 11%, respectively. Therefore, for EIS applications where only higher excitation frequencies are needed, such as determining the conductivity of an electrolyte, higher excitation voltages (e.g., 0.5 V at frequencies >100 Hz) can be applied. This gives a good range of excitation potentials for achieving higher signal-to-noise ratios for EIS measurements. However, at such higher voltages, the effect of Faradaic reactions also need to be considered when designing experiments.

## 5. Conclusions

A time-dependent EIS model was successfully developed using finite element analysis in COMSOL Multiphysics to simulate ion transport under external excitation voltage. The model was validated against experimental data for NaCl solutions.

The transient analysis demonstrates that at lower frequencies and low ion concentrations, the voltage drop across the Stern layer is the most pronounced, but it decreases significantly at higher frequencies. For higher ion concentrations, both the Stern layer and sheet resistance contribute to the overall voltage drop, even at high concentrations. This highlights the critical need to consider the impact of sheet resistance in high-frequency EIS measurements, especially in systems with low electrolyte resistance.

The time-dependent model also demonstrated the importance of simulating multiple excitation cycles to achieve a stabilized current response, providing practical insights into the number of cycles required for accurate impedance measurement. The comparison between simulation and experimental data showed good agreement at the largest part of the frequency range. Parasitic capacitance effects from the SiO_2_ layer on the silicon substrate became evident at the highest frequencies near 1 MHz, underscoring the importance of considering parasitic capacitance in silicon-based sensor designs. At lower frequencies, the experimental impedance data converged across ion concentrations, whereas the simulations exhibited frequency-dependent offsets, particularly in phase shift. This discrepancy suggests that additional physical effects, such as Faradaic processes or electrode interface dynamics, may need to be included into the model to improve its accuracy at low frequencies. Future work will aim to refine the model by addressing these limitations.

The effect of the excitation voltage amplitude has been investigated in the simulations. A critical threshold of 0.1 V was identified, beyond which significant deviations in impedance occur, particularly at lower ion concentrations and lower frequencies. The practical implication of this finding is that the excitation voltage amplitude may be selected higher than the typical default, which can improve the signal-to-noise ratio in the EIS experiments.

Overall, the developed time-dependent model offers significant insights into the processes governing EIS measurements. It provides a valuable tool for optimizing the design of EIS-based sensors and experimental parameters, demonstrating its utility in sensor development.

## Figures and Tables

**Table 1:** Overview of used symbols and their definitions.

Symbol	Details
$c_{i}$	Concentration of ion species *i* [mol/m^3^]
$D_{i}$	Diffusion coefficient of species *i* [m^2^/s]
$z_{i}$	Charge number of ion species *i*
$\lambda_{s}$	Stern layer thickness [m]
$\epsilon_{s}$	$\mathrm{Stern}\;\mathrm{layer}\;\mathrm{permittivity}\;[F/m],\;\mathrm{which}\;\mathrm{is}\;\varepsilon_{rs}\varepsilon_{0}$$,\;\mathrm{where}\;\epsilon_{rs}$ $\mathrm{and}\;\epsilon_{0}$ are the relative permittivity of the Stern layer and that of free space, respectively
$\epsilon_{liq}$	$\mathrm{Electrolyte}\;\mathrm{permittivity}\;[F/m],\;\mathrm{which}\;\mathrm{is}\;\varepsilon_{r}\varepsilon_{0}$$,\;\mathrm{where}\;\epsilon_{r}$ $\mathrm{and}\;\epsilon_{0}$ are the relative permittivity of the electrolyte and that of free space, respectively
$\sigma_{el}$	Conductivity of the electrode material [S/m],
$d_{el}$	Electrode thickness [m]
φ	Potential in the electrolyte [V]
$\varphi_{y=0}$	Potential at the working electrode [V]
$\varphi_{y=L}$	Potential at the ground electrode [V]
$\varphi_{\left(t\right)}$	Applied external voltage at the working electrode [V]
$\vec{n}$	Normal unit vector to surface
$J_{i}$	Flux of ion species *i* [mol/ (m^2^ s)]
$\vec{E}$	Electric field [V/m]

**Table 2:** Parameters and its values in the model.

Symbol	Details
$c_{bulk}$	Bulk concentration of Na^+^ and Cl^−^ ions, respectively: 1 mM, 10 mM, and 100 mM
$D_{Na}$	Diffusion coefficient of Na^+^: 1.33·10^−9^ m^2^/s [18]
$D_{Cl}$	Diffusion coefficient of Cl^−^: 2.03·10^−9^ m^2^/s [18]
$z_{i}$	+1 for Na^+^ ion and −1 for Cl^−^ ion
$\lambda_{s}$	0.5 nm [19]
$\epsilon_{liq}$	Permittivity of the electrolyte, which is equal to the product of the relative permittivity, $\epsilon_{r}=80,\;\mathrm{and}\;\mathrm{that}\;\mathrm{of}\;\mathrm{free}\;\mathrm{space},\;\epsilon_{0}=8.854\cdot 10^{-12}$ F/m
$\varphi_{\left(t\right)}$	10 mV amplitude sinusoidal excitation voltage
f	Frequency range: 0.1 Hz to 1 MHz
$k_{el}$	Electrode sheet resistance parameter. Varied range in simulations: 1·10^−13^ to 1·10^−11^ m·s. Details of the parametric analysis are given in Appendix A.
$k_{s}$	Stern layer parameter: varied range in simulations 1·10^−11^ to 1·10^−10^ m. Details of the parametric analysis are given in Appendix A.
C_par_	Parasitic capacitance from SiO_2_ layer: 0.7 nF

**Table 3:** Model dimension parameters and its values.

Symbol	Details
Electrode width, W	600 µm
Spacing between electrodes, S	600 µm
Length of one electrode finger, L_f_	6 mm
Total meander length, L_m_	65.1 mm
Electrochemical cell height, H_cell_	600 µm

**Figure A1:**
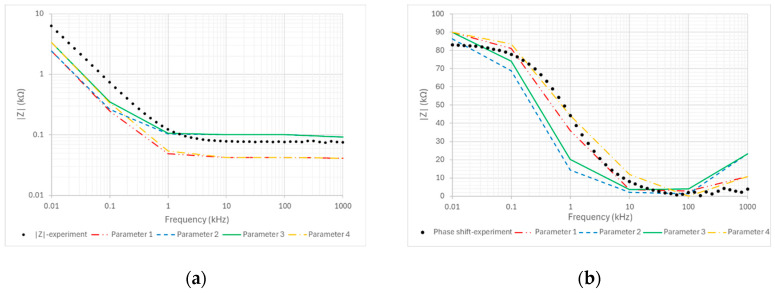
EIS plot for comparison between the experimental data and the simulation results with various k_el_ and k_s_ parameter combinations for a 100 mM ion concentration. (**a**) |Z| plot and (**b**) phase shift plot.

**Figure A2:**
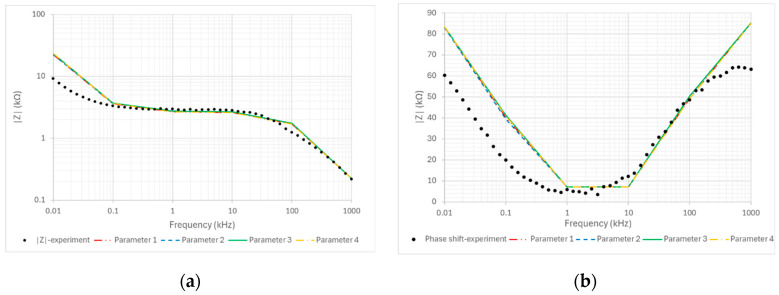
EIS plot for comparison between the experimental data and the simulation results with various k_el_ and k_s_ parameter combinations for a 100 mM ion concentration. (**a**) |Z| plot and (**b**) phase shift plot.

## Data Availability

The data presented in this study are available on request from the corresponding author.

## Appendix A

### Determination of Sheet Resistance and Stern Layer Thickness Parameters Using Parametric Analysis

The sheet resistance parameter, k_el_, and the stern layer parameter, k_s_, are both evaluated through a partial factorial analysis where a combination of both of these parameters with a wide range of values are simulated. The resulting EIS data from a simulation using these combinations are then compared with the experimental data, and the parameter combinations with the least deviation are chosen. The values for both parameters analyzed in this study are given in Table A1. The total number of combinations of k_el_ (five values) and k_s_ (four values) is 20. Among these combinations, the one with the best fit to the experimental data for 1 mM and 100 mM ion concentrations are chosen.

**Table A1:** Values of kel and ks parameters used for combinations.

Parameter	Values	Unit
k_el_	1·10^−13^, 5·10^−13^, 1·10^−12^, 5·10^−12^ and 1·10^−11^	m·s
k_s_	1·10^−10^, 5·10^−10^, 1·10^−9^and 5·10^−9^	m

The deviation of the EIS simulation results using various k_el_ and k_s_ combinations from the experimental data is analyzed by using the mean square root difference. Since the |Z| in the bode plot is in log scale, the root means square deviation of the log of the ratio between simulated, |Z|_s_ and measured, |Z|_m_, as shown in equation A1. Whereas for the deviation in the phase shift, △ϕ, simple root means square difference between simulated phase shift, ${△\phi }_{s}$, and the measured phase shift, ${△\phi }_{M}$, is evaluated, as given in Equation (A2).

(A1) $$\log deviation\;in\;|Z|=\sqrt{\sum_{i=f_{i}}^{f_{max}}\frac{{\left[{log}_{10}\;\left(\frac{{\left|Z\right|}_{s}}{{\left|Z\right|}_{M}}\right)\right]}^{2}}{N}}$$

(A2) $$Deviation\;in\;△\phi =\sqrt{\frac{\sum_{i=f_{i}}^{f_{max}}{\left({△\phi }_{s}-{△\phi }_{M}\right)}^{2}}{N}}\;$$

Here, $f_{max}$ is the maximum frequency of excitation and N is the number of frequencies applied. From the analysis of the 20 different combinations, the best fit to the experimental data is found for the k_el_ values of 1·10^−13^ and 5·10^−13^ m·s, and for k_s_ values of 1·10^−10^, and 5·10^−10^ m. The four best combinations with least deviations for 100 and 1 mM ion concentration is given in Table A2 and Table A3, respectively.

**Table A2:** Selected parameter combinations with the lowest deviation of |Z| and ϕ from the experimental values for 100 mM.

Parameter No.	Value of k_el_ (m·s)	k_s_ (m)	Log Deviation in |Z|	Deviation in △ϕ (Degrees)
Parameter 1	1·10^−13^	1·10^−10^	0.171	5.7
Parameter 2	5·10^−13^	1·10^−10^	0.171	15.24
Parameter 3	5·10^−13^	5·10^−10^	0.178	13.1
Parameter 4	1·10^−13^	5·10^−10^	0.177	4.95

**Table A3:** Selected parameter combinations with the lowest deviation of |Z| and ϕ from the experiment for 1 mM.

Parameter No.	Value of k_el_ (m·s)	k_s_ (m)	Log Deviation in |Z|	Deviation in △ϕ (Degrees)
Parameter 1	1·10^−13^	1·10^−10^	0.171	15.5
Parameter 2	5·10^−13^	1·10^−10^	0.171	15.5
Parameter 3	5·10^−13^	5·10^−10^	0.178	15.8
Parameter 4	1·10^−13^	5·10^−10^	0.177	15.7

Since the deviation analyses in Table A2 and Table A3 provide an average effect over different frequencies and can sometimes be misleading, the EIS plots for each of these parameter combinations in Table A2 and Table A3 need to be plotted to have a better understanding. These plots are shown in Figure A1 and Figure A2 for 100 and 1 mM ion concentrations, respectively.

In the case of 1 mM, all the combinations from Table A3 show almost overlapping values, so any combination from this table would provide the best fit. In the case of 100 mM, although the value of deviation (from Table A2), especially in |Z|, show close values, it appears from Figure A1 that parameter 3 has a good fit, especially in the high-frequency range. Therefore, among these combinations, parameter 3, i.e., k_el_ = 5·10^−13^ m·s and k_s_ = 5·10^−10^ m, was chosen for further analysis.
